# Cortical Plasticity Induced by Anodal Transcranial Pulsed Current Stimulation Investigated by Combining Two-Photon Imaging and Electrophysiological Recording

**DOI:** 10.3389/fncel.2019.00400

**Published:** 2019-08-29

**Authors:** Zengguang Ma, Xiaolang Du, Feifei Wang, Ran Ding, Yuanyuan Li, Aili Liu, Liangpeng Wei, Shaowei Hou, Feng Chen, Qi Hu, Cunle Guo, Qingyan Jiao, Shujing Liu, Bei Fang, Hui Shen

**Affiliations:** ^1^Laboratory of Neurobiology, School of Biomedical Engineering and Technology, Tianjin Medical University, Tianjin, China; ^2^Department of Pharmacy, Tianjin’s Clinical Research Center for Cancer, National Clinical Research Center for Cancer, Key Laboratory of Cancer Prevention and Therapy, Tianjin Medical University Cancer Institute and Hospital, Tianjin, China; ^3^Department of Anesthesiology, Tianjin Medical University General Hospital, Tianjin, China

**Keywords:** cortical plasticity, astrocyte, neuron, anodal transcranial pulsed current stimulation, two-photon calcium imaging, electrophysiological recording

## Abstract

Anodal-transcranial pulsed current stimulation (a-tPCS) has been used in human studies to modulate cortical excitability or improve behavioral performance in recent years. Multiple studies show crucial roles of astrocytes in cortical plasticity. The calcium activity in astrocytes could regulate synaptic transmission and synaptic plasticity. Whether the astrocytic activity is involved in a-tPCS-induced cortical plasticity is presently unknown. The purpose of this study is to investigate the calcium responses in neurons and astrocytes evoked by a-tPCS with different current intensities, and thereby provides some indication of the mechanisms underlying a-tPCS-induced cortical plasticity. Two-photon calcium imaging was used to record the calcium responses of neurons and astrocytes in mouse somatosensory cortex. Local field potential (LFP) evoked by sensory stimulation was used to assess the effects of a-tPCS on plasticity. We found that long-duration a-tPCS with high-intensity current could evoke large-amplitude calcium responses in both neurons and astrocytes, whereas long-duration a-tPCS with low-intensity current evoked large-amplitude calcium responses only in astrocytes. The astrocytic Ca^2+^ elevations are driven by noradrenergic-dependent activation of the alpha-1 adrenergic receptors (A1ARs), while the intense Ca^2+^ responses of neurons are driven by action potentials. LFP recordings demonstrated that low-intensity a-tPCS led to enhancement of cortical excitability while high-intensity a-tPCS resulted in diminution of cortical excitability. The results provide some evidence that the enhancement of a-tPCS-induced cortical excitability might be partly associated with calcium elevation in astrocytes, whereas the diminution of a-tPCS-induced cortical excitability might be caused by excessive calcium activity in neurons. These findings indicate that the appropriate current intensity should be used in the application of a-tPCS.

## Introduction

Transcranial electrical stimulation (tES) is a non-invasive brain stimulation technique that can modulate cortical plasticity for clinical and experimental applications. At present, the most commonly used tES method is transcranial direct current stimulation (tDCS), which has been widely used in the treatment of neurological disorders (Fregni et al., 2015; Yavari et al., 2018). It is generally assumed that the direction of cortical excitability changes depends on the polarity of current stimulation. The anodal stimulation generally enhances cortical excitability, whereas the cathodal stimulation diminishes cortical excitability (Cambiaghi et al., 2010; Fritsch et al., 2010; Kabakov et al., 2012). The use of tDCS involves the application of a constant direct current. In recent years, a novel neuromodulatory paradigm employing transcranial pulsed current stimulation (tPCS) has gained increasing attention as a promising technique to induce cortical plasticity.

In the tPCS paradigm, the continuous flow of direct current in tDCS is interrupted by a periodical inter-pulse interval. It has been shown that anodal anodal-transcranial pulsed current stimulation (a-tPCS) can exert its effects by polarity-dependent modulation of cortical activity and on-off effects of the pulses on neurons (Jaberzadeh et al., 2014). Compared to conventional tDCS, a-tPCS with short inter-pulse interval increases its efficacy for enhancement of corticospinal excitability (Jaberzadeh et al., 2014). Besides, the side effects were minimized during and after the application of a-tPCS and the participants tolerated a-tPCS better than the conventional tDCS (Jaberzadeh et al., 2014, 2015). It has been demonstrated that a-tPCS is a safe intervention and could induce acute improvement of gait and balance recovery in patients with Parkinson disease (Alon et al., 2012). Previous studies showed that tPCS could facilitate arithmetical processing on complex mathematical task and improve response time in the attention switching task (Morales-Quezada et al., 2015, 2016). The positive clinical outcomes acquired in various conditions implied that tPCS could be considered as a promising therapeutic technique in neurorehabilitation.

In the last few years, there has been an increased interest in exploring the tPCS-induced plasticity. Jaberzadeh et al. (2014) concluded that a-tPCS altered cortical excitability by a combination of tonic and phasic effects. Morales-Quezada et al. (2014) verified that tPCS could modulate inter-hemispheric coherence of brain oscillatory activity and enhance functional connectivity. Recent studies showed that tPCS can modulate brain oscillation in a frequency-specific manner (Thibaut et al., 2017; Vasquez et al., 2017; Singh et al., 2019). In these studies, researchers mainly used electroencephalographic (EEG) recording to reveal the neuronal activity in the brain. Multiple studies have shown that astrocytes could play a critical role in the modulation of synaptic plasticity (Chung et al., 2015; Haydon and Nedergaard, 2015; Papouin et al., 2017). Therefore, recording of astrocytic activity during tPCS is also important to better understand the mechanism of action.

Astrocytes are not electrically excitable, however, they display their excitability through variations in intracellular calcium signals (Papouin et al., 2017). In brain tissues, excitatory synapses are usually approached or surrounded by fine astrocytic protrusions (Heller and Rusakov, 2015). Intracellular Ca^2+^ elevation in astrocytes can trigger the release of various gliotransmitters, which can act on neurons to regulate synaptic transmission and plasticity (Perea et al., 2009; Di Castro et al., 2011; Panatier et al., 2011; Guerra-Gomes et al., 2018). Two-photon calcium imaging is a powerful means for monitoring the calcium changes of neurons and astrocytes in the brain at high resolution. By using this technique, neuronal and astrocytic Ca^2+^ dynamics in the cortex can be monitored to reveal the possible role of these cells in induction of cortical plasticity. Based on two-photon calcium imaging, Takata et al. (2011) provided evidence that astrocytic activity was involved in cortical plasticity induced by electrical stimulation of the nucleus basalis of Meynert. It has been demonstrated that tDCS-induced cortical plasticity was associated with astrocytes, which display large-amplitude Ca^2+^ surges during tDCS (Monai et al., 2016; Monai and Hirase, 2018). Compared to tDCS, pulsed stimulation protocol can modulate cortical activity in a frequency-dependent manner (Vasquez et al., 2016; Singh et al., 2019). Up to now, whether the astrocytic activity is involved in a-tPCS-induced cortical plasticity is still largely unknown.

Anodal-transcranial pulsed current stimulation delivered at low frequency with short inter-pulse interval could induce reliable enhancement of corticospinal excitability (Jaberzadeh et al., 2014, 2015). The similar frequency and inter-pulse interval were used in this study. In addition, current intensity is an important parameter in determining the modulatory effects of tPCS. Morales-Quezada et al. (2014) demonstrated that tPCS has an intensity-dependent facilitatory effect on interhemispheric connectivity. The change in current intensity of electrical stimulation resulted in a corresponding modulation in the strength and duration of the stimulation after-effects (Nitsche and Paulus, 2000). A previous study showed that the enhancement of tDCS intensity is not always accompanied by increased efficacy, but might even change the direction of effects (Batsikadze et al., 2013). At present, no study has explored the effects of a-tPCS with different current intensities on cellular responses. The purpose of this study is to investigate the cellular responses to a-tPCS with different stimulation intensities, and improve the understanding of the mechanisms underlying a-tPCS-induced cortical plasticity. We used two-photon calcium imaging to record the calcium changes of neurons and astrocytes in mouse somatosensory cortex. Electrophysiological recording of local field potential (LFP) was used to assess the cortical plasticity evoked by a-tPCS. We hypothesized that the a-tPCS-induced cortical plasticity might be associated with the calcium changes in astrocytes.

## Materials and Methods

### Animal Preparation

All experimental procedures were performed with approval from the Animal Care and Use Committee of Tianjin Medical University and were in accordance with the National Institutes of Health Guide for the Care and Use of Laboratory Animals.

Thirty-five male C57BL/6 mice aged 8∼12 weeks were used in this study. The animals were housed under a 12 h light/12 h dark cycle (lights on at 7:00 am) with free access to food and water. Initial anesthesia was induced with 2% isoflurane in pure oxygen using a gas anesthesia system (Model 3000, Matrx, United States). The animals were anesthetized with isoflurane (0.8–1.5% during surgery, 0.5–0.8% during recording) in pure oxygen and the body temperature was kept at approximately 37° with a heating pad. To prevent drying, the animal eyes were covered by ophthalmic ointment. After removing the skin above the skull, a custom-made plastic chamber was cemented onto the skull with cyanoacrylate glue (UHU, Germany) over the right primary somatosensory cortex according to stereotaxic coordinates. A circular craniotomy (2–3 mm in diameter) was performed to expose the forepaw region of the primary somatosensory cortex (S1) [anterior-posterior (AP) 0.5 mm and medio-lateral (ML) 2.25 mm] and the dura mater of the cortex was removed. The recording chamber was perfused with artificial cerebral spinal fluid (ACSF) (composition in mM: 125 NaCl, 4.5 KCl, 26 NaHCO_3_, 1.25 NaH_2_PO_4_, 2 CaCl_2_, 1 MgCl_2_ and 20 Glucose, pH 7.4 when bubbled with 95% O_2_ and 5% CO_2_).

For two-photon calcium imaging, the Ca^2+^ sensitive fluorescent indicator Oregon Green 488 BAPTA-1 (OGB-1) and the red astrocyte-specific dye sulforhodamine 101 (SR101) were used. OGB-1 AM (Molecular Probes-Invitrogen, United States) was dissolved in DMSO with 20% Pluronic F-127 (Sigma–Aldrich, United States) and diluted in ACSF to a final concentration of 1 mM. By using multi-cell bolus loading technique (Stosiek et al., 2003; Garaschuk et al., 2006), we delivered the dye to the cortical cells. A glass micropipette with a resistance of 3–4 MΩ was filled with the dye mixture of 1 mM OGB-1 AM and 100 μM SR101 (Molecular Probes-Invitrogen, United States). The micropipette was inserted into the cortex at a depth of ∼150–230 μm. Dye injections within this depth range would result in almost spherical stained areas covering cortical layers 1–3. The dye mixture was pressure (2.5 min, 400–500 mbar) ejected into the tissue under two-photon imaging. The pressure was monitored by a digital manometer (AZ 8230, AZ Instrument Corporation, China). After dye injection, the exposed cortex was covered with low melting point agarose (1.5% w/v in ACSF) and partly sealed with a thin glass coverslip (diameter, 3 mm; thickness, 0.12 mm). Before recording, we allowed 60 min for loading so as to obtain a stable fluorescence signal in stained cells. For electrophysiological recording, a screw electrode fixed to the skull was used as the reference electrode.

### *In vivo* Two-Photon Imaging

*In vivo* imaging of OGB-1 and SR101 was performed using a two-photon microscope (A1R MP, Nikon, Japan), and a femtosecond Ti:sapphire laser (MaiTai DeepSee, Spectra-Physics, Germany) with a water-immersion objective (40×, 0.8 NA, Nikon, Japan). The wavelength of excitation light was set to 900 nm. Image acquisition was acquired using Nikon NIS-Elements AR software. The cortical area was imaged at a 30-Hz frame rate with high resolution (512 × 512 pixels). The time-lapse images from the green (emission collected at 500–550 nm) and red (emission collected at 601–657 nm) channels were simultaneously obtained. For short-duration a-tPCS (stimulus duration, 2 s), each recording trial lasted for 70 s containing 10 s before the stimulus onset. For long-duration a-tPCS (stimulus duration, 5 min), each recording trial lasted for 8 min containing 1 min before the stimulus onset.

### *In vivo* Electrophysiological Recordings

Extracellular recordings of LFP were performed with an Axon 200B patch-clamp amplifier (Molecular Devices, United States) and Digidata 1550B interface (Molecular Devices, United States). A glass micropipette filled with ACSF was mounted in an electrode holder and attached to the headstage. The recording electrode had a resistance of 2–4 MΩ. The electrode was inserted to a depth of 180–220 μm at an insertion angle of 30° in the cortex using a micromanipulator system. After insertion, it takes at least 1 h to make the evoked LFP stable. Electrophysiological data were captured at a 10-kHz sampling rate and filtered at 2 kHz. Each recording session lasted 50 s and consisted of five stimuli with an inter-stimulus interval of 10 s. The recording session was performed every 10 min.

### Sensory and Anodal-Transcranial Pulsed Current Stimulation

For sensory stimulation, electrical stimulation (duration 1 ms, intensity 0.3–0.4 mA) was delivered to the forepaw contralateral to cortical exposure through a metal clip connected to a constant current stimulator (Model 2100, AM-system, United States). The electrical stimulation was applied at a frequency of 0.1 Hz for 50 s, with an interval of 10 min between different recording sessions.

For a-tPCS, the stimulating electrode was made of silver wire and a glass micropipette with a broken tip filled with ACSF. The impedances of electrodes were between 50 and 80 KΩ. The glass micropipette was positioned on the 1.5% agarose above the somatosensory cortex. The recording chamber was perfused with ACSF. A stainless steel needle inserted into the dorsal neck muscle was used as the return electrode. Rectangular pulses of anodal current generated by a stimulus isolator (Model 2100, AM-system, United States) were used for the a-tPCS. According to previous findings, the frequency and pulse width for a-tPCS were set to be 2 Hz and 450 ms, respectively (Jaberzadeh et al., 2014). Different current intensities (0.1–0.35 mA) were used for unveiling the intensity-dependent effects on cortical response. In our experiments, we first explored the calcium transients in neurons and astrocytes evoked by short-duration (2 s) a-tPCS. Then, we mainly investigated the cellular calcium changes and cortical plasticity induced by long-duration (5 min) a-tPCS.

### Drug Application

For pharmacological experiments, reagents were dissolved in ACSF and applied on the cranial window from 30 min preceding imaging. Tetrodotoxin (TTX, 2 μM, Tocris) was used to block sodium channel. DL-2-amino-5-phosphonovaleric acid (AP-5, 50 μM, Tocris) and 2,3-dihydroxy-6-nitro-7-sulfamoyl-benzo(f)quinoxaline-2,3-dione (NBQX, 20 μM, Tocris) were used to block NMDA-type and AMPA-type ionotropic glutamate receptors, respectively. Prazosin hydrochloride (200 μM, Sigma–Aldrich) was used to block alpha-1 adrenergic receptors (A1ARs).

### Data Analysis

**FIGURE 1:**
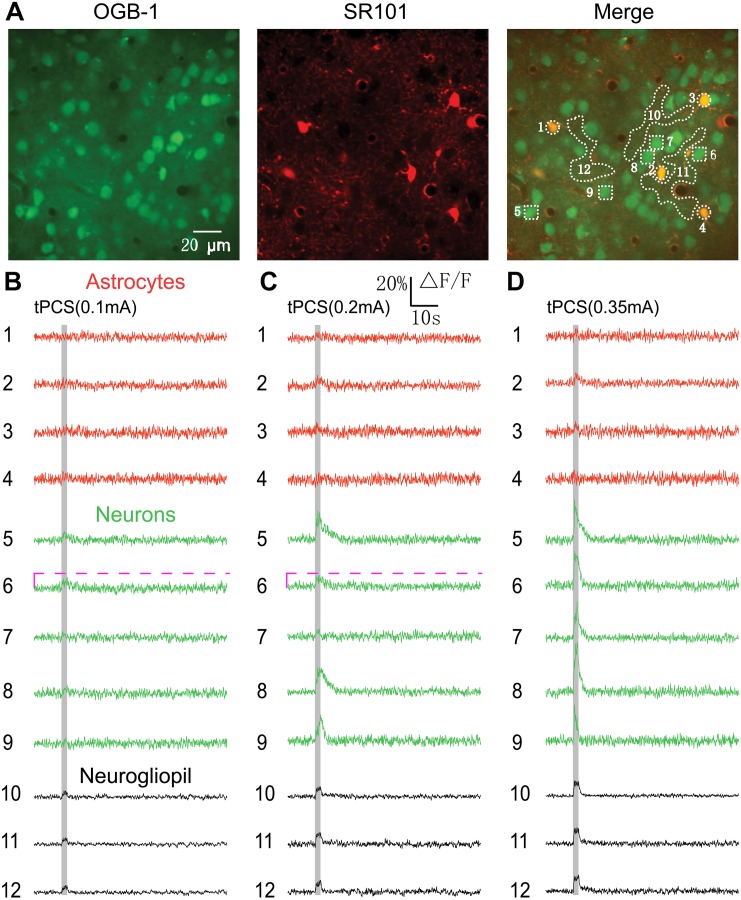
Effects of 2 s a-tPCS (frequency, 2 Hz; pulse width, 450 ms) with different current intensities on calcium changes in astrocytes, neurons and neurogliopil regions. **(A)** *In vivo* image of a patch of cortex 190 μm below the pial surface loaded with the calcium indicator OGB-1 and an astrocyte marker, SR101. The size of the field-of-view was ∼160 μm × 160 μm. From left to right, the panels show cells loaded with Ca^2+^ indicator OGB-1 (green), astrocytic marker SR101 (red), and merged OGB-1 and SR101. ROIs were placed on neuronal somata, astrocyte somata and neurogliopil regions. Circles indicate astrocyte somata, the squares represent neuronal somata and the irregular contours delineate the neurogliopil regions. **(B–D)** Time courses of 2 s a-tPCS induced Ca^2+^ responses (ΔF/F) in the astrocytes (1–4), neurons (5–9) and neurogliopil (10–12) outlined in the right panel of **(A)**. The current intensities were 0.1 mA **(B)**, 0.2 mA **(C)**, and 0.35 mA **(D)**. Each trace lasted for 70 s with 10-s pre-stimulus baseline. Gray area denotes the stimulus duration (2 s). Numbers before each trace correspond to the cells and neurogliopil regions marked in the merged image of panel **(A)**. Dashed pink line represents activity detection threshold (10%).

**FIGURE 2:**
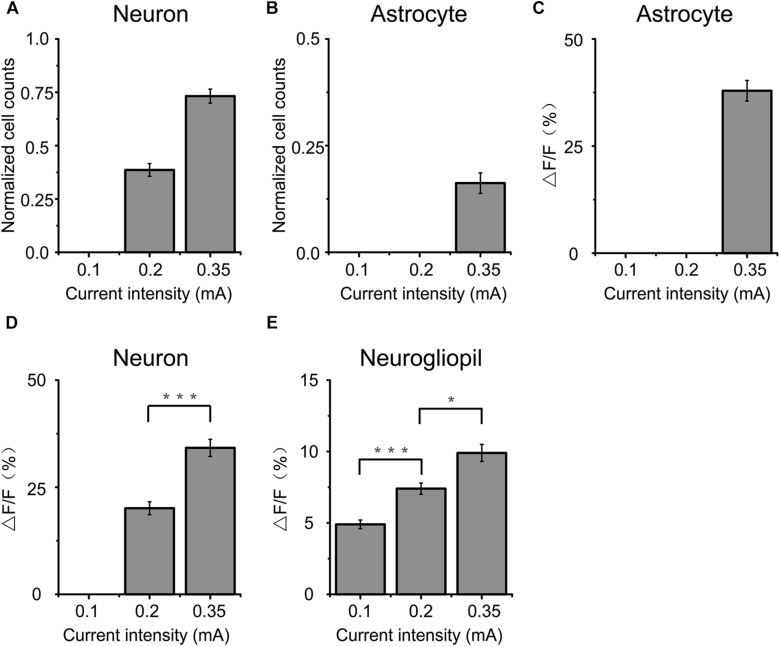
Comparison of cortical responses to 2 s a-tPCS with different current intensities. **(A,B)** Percentage of active (response amplitude > 10% ΔF/F was considered as significant response) cells in response to a-tPCS at varying current intensities. **(C–E)** Bar graph comparing amplitudes of calcium signals in active astrocytes **(C)**, neurons **(D)**, and neurogliopil regions **(E)**, respectively. Statistical significance was tested with two-sample *t*-test. ^∗^*P* < 0.05 and ^∗∗∗^*P* < 0.0001. Error bars indicate SEM.

All data analyses were performed using custom-written software in LabVIEW 2014 (National Instruments), NIS-Elements AR (Nikon), Clampfit10.6 (Axon) and Matlab 2014a (MathWorks). Astrocytes were identified by OGB-1 AM and SR101 co-labeling. To select the regions of interest (ROIs), the time-lapse images of OGB-1 fluorescence during one recording trial were averaged. For calcium analysis, ROIs were outlined manually based on fluorescence intensity and cell body morphology on the averaged image. While the cell bodies of neurons and astrocytes are identifiable in the merged image, the areas between the cell bodies are also loaded with OGB-1. The neurogliopil region contains the processes of surrounding neurons and astrocytes. The contour of neurogliopil region was visually identified and outlined manually on the basis of the image intensity. To extract the Ca^2+^ fluorescence changes from the image data, the pixel values within each ROI were averaged. Ca^2+^ signals over time are presented as the relative change in fluorescence (ΔF/F), where the ΔF is the difference from the pre-stimulus baseline mean and F is the pre-stimulus baseline mean. For multi-cell bolus loading, one common problem in population Ca^2+^ imaging is that somatic fluorescence can often be contaminated by adjacent neurogliopil (Garaschuk et al., 2006; Histed et al., 2009) (see Figures 1B–D). The cell bodies may contain components of the neurogliopil signal below or above them because the two-photon imaging plane is not infinitely thin (Garaschuk et al., 2006; Histed et al., 2009). In our experiments, the craniotomy was partly sealed with a glass coverslip, leaving the lateral side of the craniotomy open for insertion of a glass electrode for electrical stimulation. This method resulted in small brain pulsations during imaging due to the respiration of the animal. Therefore, cells that are above or below the imaging plane contain components of the neurogliopil signal below or above them. However, neurogliopil contribution in somatic fluorescence was less than the amplitude of adjacent neurogliopils’ signal. Based on the average amplitude of adjacent neurogliopils’ signals, we classified cells as activated when their fluorescence intensity (ΔF/F) exceeded 10% (see Figure 2E) for most cases and 16% (see Figure 6F) for 5-min a-tPCS with 0.35-mA intensity. The similar threshold method has been used in a previous study (Histed et al., 2009). In order to reduce the effects of cortical pulsation on fluorescence signals, the fluorescence trace was low-pass filtered (cutoff frequency 9). Amplitude of Ca^2+^ signal was obtained after smoothing the trace using a moving average filter. Onset latency of astrocytic Ca^2+^ surge was the time interval between the stimulation onset and the time when ΔF/F reached 10% of peak amplitude of Ca^2+^ surge.

For electrophysiological recording, the slope of the evoked LFP was measured. The slope of the LFP was calculated as described previously (Takata et al., 2011; Monai et al., 2016) and was summarized briefly here. The initial negative deflection of the evoked LFP was isolated, and then the slope from 20–80% of the peak-to-peak amplitude of the negative deflection was calculated (see Figure 8C). The slopes used in our analysis were averaged across five trials in one recording session.

Data are expressed as the mean ± standard error of the mean (SEM). For comparisons of two sample means, two-sample *t*-tests (Figures 2D,E, 6C–F) and Mann–Whitney tests (Figure 8F) were carried out with SPSS software (SPSS Inc., United States). *P* < 0.05 was considered statistically significant for all the tests.

## Results

### Properties of Astrocytic, Neuronal, and Neurogliopil Calcium Responses to Short-Duration a-tPCS With Different Current Intensities

In this study, we first used two-photon imaging to investigate the a-tPCS-induced (stimulus duration, 2 s) calcium changes in layer 2/3 of somatosensory cortex in anesthetized mice. Cells in cortical layers 1–3 were labeled with the calcium indicator OGB-1 and the astrocyte marker SR101. An area of 160 μm × 160 μm was imaged (Figure 1A). The merged image showed neurons in green and astrocytes in yellow at a cortical depth of 190 μm. Traces of Figures 1B–D displayed the Ca^2+^ dynamics in astrocytes, neurons and neurogliopil regions during 2 s a-tPCS with different current intensities. In Figure 1B, both neurons and astrocytes showed no obvious fluorescence changes by low-intensity (0.1 mA) a-tPCS. The neurogliopil regions selected in Figure 1A typically showed synchronized and small-amplitude Ca^2+^ responses. In Figure 1C, some neuronal somata showed synchronized and small-amplitude Ca^2+^ responses (trace number 5, 8, and 9) while other neuronal somata (trace number 6 and 7) were inactive (response amplitude > 10% ΔF/F was considered as significant response change) by 0.2 mA a-tPCS. The neurogliopil signals were obvious and the astrocyte somata were inactive. In Figure 1D, synchronized and large-amplitude Ca^2+^ responses were induced in neuronal somata by high-intensity (0.35 mA) a-tPCS. The neurogliopil regions also displayed synchronized and obvious Ca^2+^ signals after stimulus onset. However, the astrocyte somata were not active.

The data from 15 mice were used to perform analysis (Figure 2). We found that both neurons and astrocytes were not activated (response amplitude > 10% ΔF/F was considered as significant response) by 2 s a-tPCS with current intensity of 0.1 mA. When the current intensity was increased from 0.2 mA to 0.35 mA, the proportions of neurons activated by a-tPCS were increased from 38.6 ± 3.0% to 73.2 ± 3.3% (Figure 2A). As shown in Figure 2D, the mean amplitude of the neuronal response to 0.35 mA a-tPCS was significantly higher than that of the neuronal response to 0.2 mA a-tPCS (34.2 ± 2.1% vs. 20.1 ± 1.5%; two-sample *t*-test, *P* = 8.7E–6, *n* = 15). For astrocytes, it was still not activated when the current intensity was 0.2 mA (Figure 2B). The proportion of astrocytes activated by a-tPCS was 16.2 ± 2.4% when the current intensity was increased to 0.35 mA (Figure 2B). The mean amplitude of the astrocytic response to 0.35 mA a-tPCS was 37.9 ± 2.4% (Figure 2C). As can be seen in Figure 2E, the mean amplitude of the neurogliopil response at 0.2 mA was significantly larger than that of the neurogliopil response at 0.1 mA (7.4 ± 0.4% vs. 4.9 ± 0.3%; two-sample *t*-test, *P* = 2.9E–5, *n* = 15). The mean amplitude of the neurogliopil response at 0.35 mA was significantly larger than that of the neurogliopil response at 0.2 mA (9.9 ± 0.6% vs. 7.4 ± 0.4%; two-sample *t*-test, *P* = 0.0011, *n* = 15). These results indicated that 2 s a-tPCS with current intensity of 0.1 mA cannot evoke significant calcium responses (response amplitude > 10% ΔF/F was considered as significant response) in neurons and astrocytes, whereas 0.35 mA a-tPCS can reliably evoke calcium response in large numbers of neurons.

### Astrocyte Activation by Long-Duration a-tPCS With Low-Intensity Current

**FIGURE 3:**
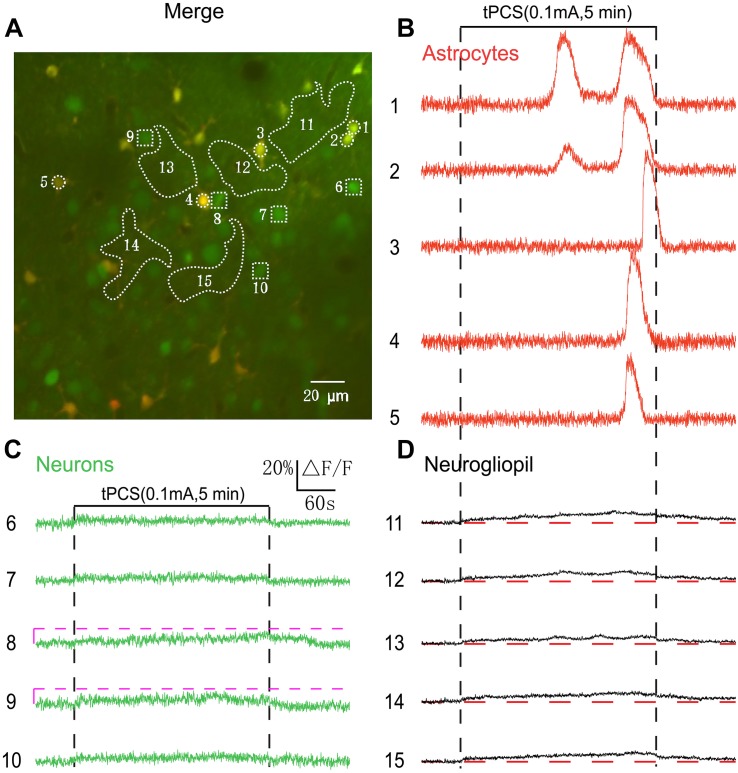
Effects of low-intensity a-tPCS (stimulus duration, 5 min; current intensity, 0.1 mA; frequency, 2 Hz; pulse width, 450 ms) on calcium changes in astrocytes, neurons and neurogliopil regions of layer 2/3. **(A)** *In vivo* image of a patch of cortex 200 μm below the pial surface loaded with the calcium indicator OGB-1 and an astrocyte marker, SR101. The size of the field-of-view was ∼210 μm × 210 μm. ROIs were placed on neuronal somata, astrocyte somata, and neurogliopil regions. Circles indicate astrocyte somata, the squares represent neuronal somata and the irregular contours delineate the neurogliopil regions. **(B–D)** Time courses of 5 min a-tPCS induced Ca^2+^ responses (ΔF/F) in the astrocytes (1–5), neurons (6–10), and neurogliopil regions (11–15) outlined in panel **(A)**. Each trace lasted for 8 min with 1-min pre-stimulus baseline. Numbers before each trace correspond to the cells and neurogliopil regions marked in the merged image of panel **(A)**. Dashed pink line represents activity detection threshold (10%).

By using 2 s a-tPCS, our results showed that low-intensity (0.1 mA) a-tPCS can evoke small-amplitude Ca^2+^ responses only in neurogliopil but not in somata of neurons and astrocytes. It has been demonstrated that long-duration a-tPCS can induce cortical plasticity. The difference between cortical responses evoked by a-tPCS with different stimulus durations is still unknown. Therefore, 5 min a-tPCS was used to investigate the cellular activity in the following experiments. The merged image of Figure 3A shows double labeling of a 210-by-210 μm square patch of cortex located 200 μm (layer 2/3) below the cortical surface. Figures 3B–D shows the Ca^2+^ responses of the somata and neurogliopil marked in Figure 3A. We found that no obvious changes occurred in fluorescence intensity of neuronal somata by 5 min a-tPCS (Figure 3C). This result was similar to the result of 2 s a-tPCS (Figure 1B). In contrast, non-synchronized and large-amplitude Ca^2+^ surges were found in most astrocytes (Figure 3B). During up to 8 min recording, the calcium elevation in some astrocyte somata even appeared twice (trace number 1 and 2). After stimulus onset, the Ca^2+^ signals of neurogliopil maintained a lower but persistent calcium elevation than baseline level (Figure 3D). These results indicated that the 5 min a-tPCS with low intensity (0.1 mA) could induce calcium elevations in astrocyte somata and neurogliopil regions but not in neuronal somata in layer 2/3.

**FIGURE 4:**
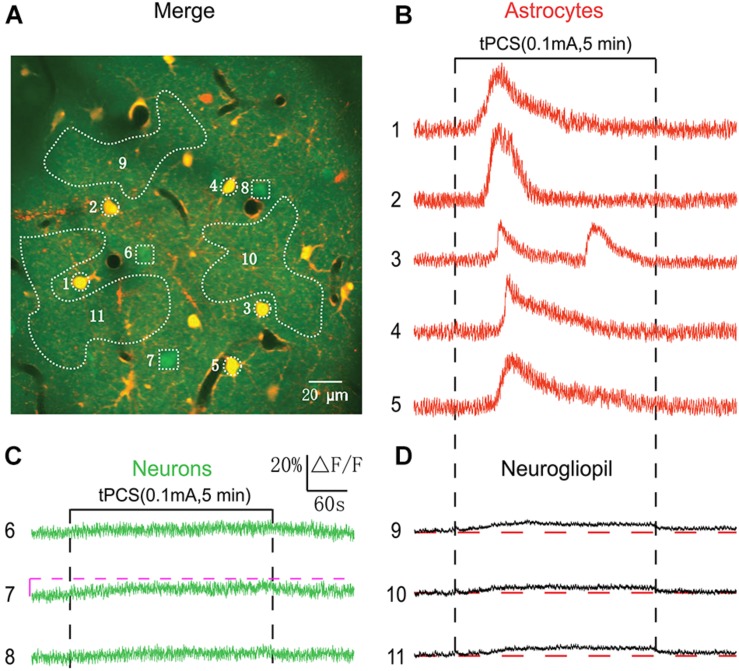
Effects of low-intensity a-tPCS (stimulus duration, 5 min; current intensity, 0.1 mA; frequency, 2 Hz; pulse width, 450 ms) on calcium changes in astrocytes, neurons and neurogliopil regions of layer 1. **(A)** *In vivo* image of a patch of cortex 46 μm below the pial surface loaded with the calcium indicator OGB-1 and an astrocyte marker, SR101. The size of the field-of-view was ∼210 μm × 210 μm. **(B–D)** Time courses of 5 min a-tPCS induced Ca^2+^ responses (ΔF/F) in the astrocytes (1–5), neurons (6–8), and neurogliopil regions (9–11) outlined in panel **(A)**. Each trace lasted for 8 min with 1-min pre-stimulus baseline. Numbers before each trace correspond to the cells and neurogliopil regions marked in the merged image of panel **(A)**. Dashed pink line represents activity detection threshold (10%).

Anatomically, cortical layer 1 has more astrocytes than layer 2/3, while layer 2/3 contains mostly neurons. In Figure 3, low-intensity a-tPCS induced large calcium elevations in astrocyte somata in layer 2/3 which is dominated with neuronal somata. It is not clear whether a-tPCS can induce calcium elevations in astrocyte somata in layer 1 which is dominated with astrocyte somata. In Figure 4A, the merged image with a field of view of 210 μm × 210 μm shows astrocytes and neurons at the cortical depth of 46 μm (layer 1). As was shown, most of the loaded cells were astrocytes. Figures 4B–D shows the time courses of a-tPCS-induced Ca^2+^ responses of the cell bodies and neurogliopil regions presented in Figure 4A. In layer 1, most astrocytes displayed non-synchronized and large-amplitude Ca^2+^ surges after stimulus onset, whereas neurons still did not show obvious changes in fluorescence intensity. Besides, the lower and sustained calcium signals after stimulus onset were also observed in different neurogliopil regions.

### Neuronal and Astrocytic Activation by Long-Duration a-tPCS With High-Intensity Current

**FIGURE 5:**
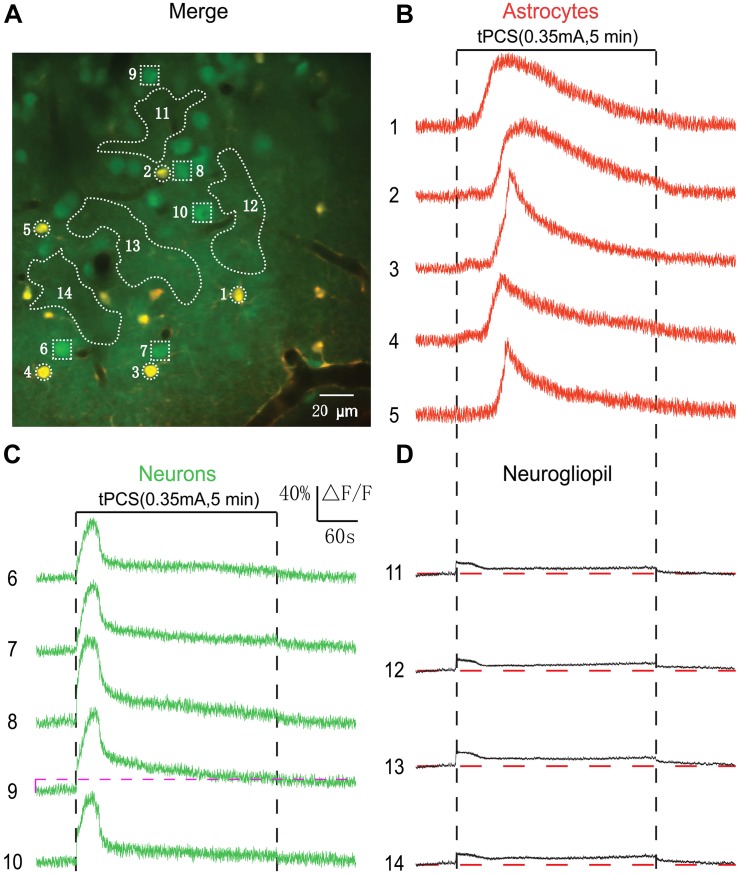
Effects of high-intensity a-tPCS (stimulus duration, 5 min; current intensity, 0.35 mA; frequency, 2 Hz; pulse width, 450 ms) on calcium changes in astrocytes, neurons and neurogliopil regions. **(A)** *In vivo* image of a patch of cortex 160 μm below the pial surface loaded with the calcium indicator OGB-1 and an astrocyte marker, SR101. The size of the field-of-view was ∼210 μm × 210 μm. **(B–D)** Time courses of 5 min a-tPCS induced Ca^2+^ responses (ΔF/F) in the astrocytes (1–5), neurons (6–10), and neurogliopil regions (11–14) outlined in panel **(A)**. Each trace lasted for 8 min with 1-min pre-stimulus baseline. Numbers before each trace correspond to the cells and neurogliopil regions marked in the merged image of panel **(A)**. Dashed pink line represents activity detection threshold (16%).

By using low-intensity a-tPCS, we did not record significant Ca^2+^ responses in most of the neurons. Therefore, the current intensity we used here is lower than the neuronal activation threshold of most neurons. It has been demonstrated that the a-tPCS with current intensity of 0.35 mA can reliably induce Ca^2+^ responses in a large number of neurons (Figure 1D). In the following experiments, we investigated the cellular responses evoked by long-duration a-tPCS with current intensity of 0.35 mA (Figure 5). Figure 5A shows representative two-photon image of the somatosensory cortex layer 2/3. After stimulus onset, the astrocyte somata exhibited larger Ca^2+^ surges (Figure 5B) than those evoked by low-intensity a-tPCS (Figures 3B, 4B). Notably, there is an initial small increase in fluorescence intensity before Ca^2+^ surge. This small fluctuation was caused by the neurogliopil response (see Materials and Methods). The subsequent large increase was the Ca^2+^ surge of astrocyte soma. Additionally, and importantly, the neurons showed synchronized and large-amplitude Ca^2+^ responses (Figure 5C), which were not observed in studies by low-intensity a-tPCS (Figures 3C, 4C). The neurogliopil response exhibited a stimulus-locked calcium increase, and then gradually decreased to a low level which was maintained throughout the stimulation period. These results proved that 5 min a-tPCS with high intensity (0.35 mA) could elicit large-amplitude Ca^2+^ signals in both neurons and astrocytes.

### Comparison of Cortical Responses to Long-Duration a-tPCS With Different Current Intensities

**FIGURE 6:**
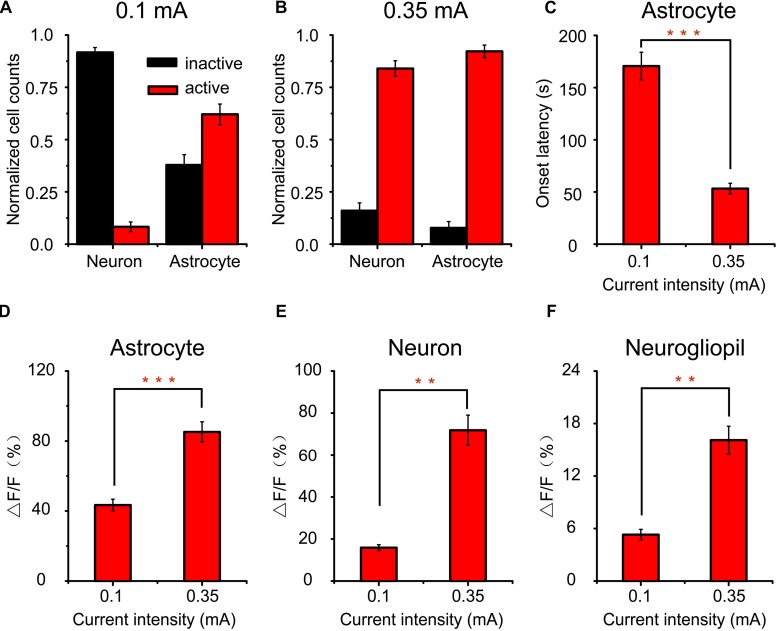
Comparison of cortical responses to long-duration a-tPCS with different current intensities. **(A,B)** Bar graph comparing the percentages of active and inactive cells in response to a-tPCS at low **(A)** and high intensity **(B)**, respectively. **(C)** Bar graph comparing onset latencies of calcium signals in active astrocytes. **(D–F)** Bar graph comparing amplitudes of calcium signals in active astrocytes **(D)**, neurons **(E)**, and neurogliopil regions **(F)**, respectively. Statistical significance was tested with two-sample *t*-test. ^∗∗^*P* < 0.001 and ^∗∗∗^*P* < 0.0001. Error bars indicate SEM.

To investigate the differences between cortical responses evoked by low- and high-intensity a-tPCS, the proportions of activated cells were compared and shown in Figures 6A,B. For low-intensity (0.1 mA) a-tPCS, about 91.4 ± 1.2% of neurons showed no significant response (response amplitude > 10% ΔF/F was considered as significant response) and only about 8.6 ± 1.2% of neurons (*n* = 7 mice) showed significant response (Figure 6A). However, this is not the case for astrocytes. The percentage of astrocytes displaying significant response was up to 62.1 ± 4.9% and the remaining 37.9 ± 4.9% of astrocytes (*n* = 7 mice) showed no significant response (Figure 6A). That means the activated cells during low-intensity a-tPCS were mainly astrocytes but not neurons. For high-intensity (0.35 mA) a-tPCS, approximately 83.5 ± 1.4% of neurons exhibited significant response (response amplitude > 16% ΔF/F was considered as significant response) and about 16.5 ± 1.4% of neurons (*n* = 6 mice) were inactive (Figure 6B). The percentage of activated astrocytes was up to 92.2 ± 3% and only about 7.8 ± 3% of astrocytes (*n* = 6 mice) were inactive (Figure 6B). These results showed that long-duration a-tPCS with high-intensity current can activate both neurons and astrocytes, whereas long-duration a-tPCS with low-intensity current mainly activate astrocytes. Among activated astrocytes, the onset latency of astrocyte response to high-intensity a-tPCS was significantly shorter than that of astrocyte response to low-intensity a-tPCS (Figure 6C: 53.3 ± 5.1 s vs. 170.6 ± 13.3 s, two-sample *t*-test, *P* = 4.4E–5, *n* = 7 for low intensity and *n* = 6 for high intensity), suggesting that high-intensity a-tPCS can activate astrocytes quickly. Further analysis revealed that the astrocytic Ca^2+^ responses induced by high-intensity a-tPCS had significantly higher amplitudes than those induced by low-intensity a-tPCS (Figure 6D: 85.2 ± 5.8% vs. 43.4 ± 3.4%, two-sample *t*-test, *P* = 4.7E–5, *n* = 7 for low intensity and *n* = 6 for high intensity). Among activated neurons, the amplitudes of neuronal Ca^2+^ responses evoked by high-intensity a-tPCS were significantly larger than those of neuronal Ca^2+^ responses evoked by low-intensity a-tPCS (Figure 6E: 71.8 ± 7.2% vs. 15.9 ± 1.4%, two-sample *t*-test, *P* = 4.3E–4, *n* = 7 for low intensity and *n* = 6 for high intensity). In addition, the average magnitudes of Ca^2+^ responses in neurogliopil regions evoked by high-intensity a-tPCS were also significantly higher than those of Ca^2+^ responses in neurogliopil regions evoked by low-intensity a-tPCS (Figure 6F: 16.1 ± 1.6% vs. 5.3 ± 0.6%, two-sample *t*-test, *P* = 5.0E–4, *n* = 7 for low intensity and *n* = 6 for high intensity).

### Origin of Calcium Signals in Neurons and Astrocytes Induced by Long-Duration a-tPCS

**FIGURE 7:**
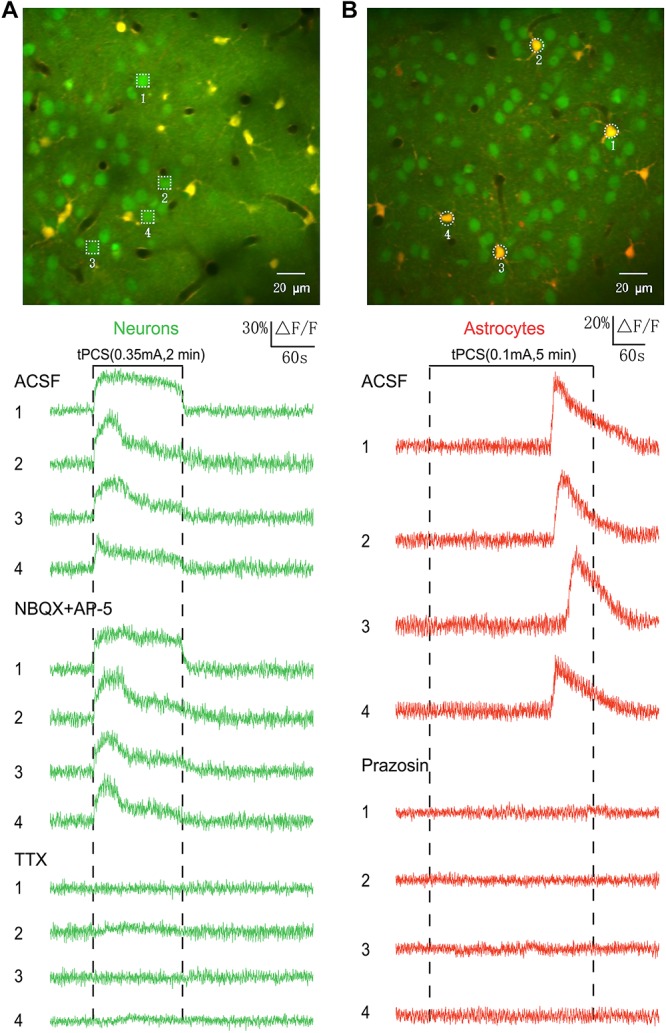
Origin of calcium signals in neurons and astrocytes induced by long-duration a-tPCS. **(A)** The intense calcium responses in neurons were not blocked by the local application of AP-5 (50 μM, 30 min before imaging) and NBQX (20 μM, 30 min before imaging), whereas they were blocked by the application of TTX (2 μM, 30 min before imaging). Each trace lasted for 6 min with 1-min pre-stimulus baseline. The experiments were performed in three animals. **(B)** The calcium signals in astrocytes were blocked by local application of prazosin (200 μM, 30 min before imaging). Each trace lasted for 8 min with 1-min pre-stimulus baseline. The experiments were performed in three animals.

To determine the induction mechanisms of calcium signals in neurons and astrocytes, pharmacological experiments were conducted (Figure 7). We used AP-5 (50 μM, Tocris) and NBQX (20 μM, Tocris) to block excitatory glutamatergic transmission and measured the effects of stimulation after drug application (Figure 7A). In our experiments, the fluorescence intensity (OGB-1) of cells would become weaker after 5 min a-tPCS with high-intensity current. However, the stimulation with 1–2 min has almost no influence on the fluorescence intensity of cells. For comparing and analyzing the effect of glutamate receptor antagonists AP-5 and NBQX on neuronal responses, the 2 min a-tPCS with high-intensity current was used. The intense calcium responses in neurons were not blocked by the local application of AP-5 and NBQX, indicating that the intense calcium responses in neurons are not induced by excitatory synaptic transmission. The neuronal responses were blocked by the application of sodium channel blocker TTX (2 μM, Tocris), suggesting that the intense Ca^2+^ responses of neurons are driven by action potentials. The experiments were performed in three animals.

To investigate the origin of calcium signals in astrocytes, the alpha 1-adrenergic antagonist prazosin (200 μM, Tocris) was used (Figure 7B). The calcium signals in astrocytes were blocked by local application of prazosin, indicating that the astrocytic Ca^2+^ elevations are driven by noradrenergic-dependent activation of the A1ARs. The experiments were performed in three animals.

### Cortical Plasticity Induced by Long-Duration a-tPCS

**FIGURE 8:**
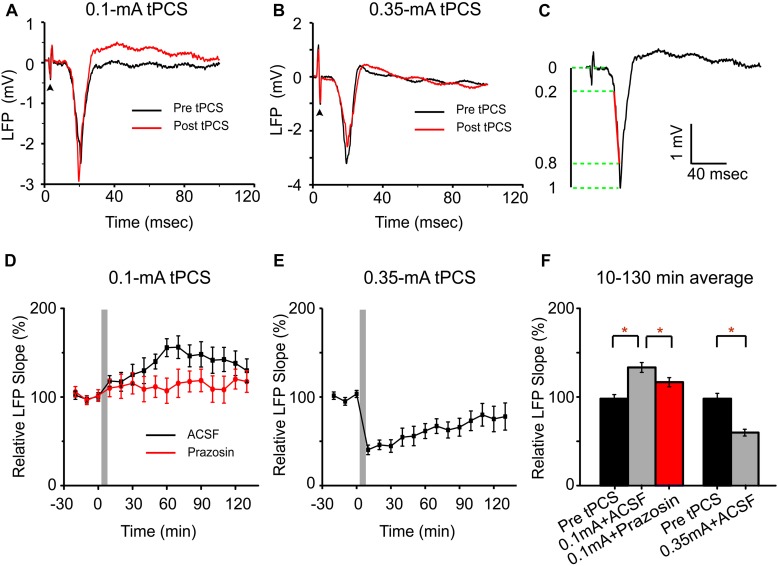
Low-intensity a-tPCS (stimulus duration, 5 min; current intensity, 0.1 mA; frequency, 2 Hz; pulse width, 450 ms) enhances LFP response in somatosensory cortex evoked by sensory stimulation while high-intensity a-tPCS (stimulus duration, 5 min; current intensity, 0.35 mA; frequency, 2 Hz; pulse width, 450 ms) diminishes it. **(A)** Representative LFP signals in response to forepaw stimulation before (black) and after (red) low-intensity a-tPCS. During 50-s recording session, forepaw stimulation was applied every 10 s, which triggered a reliable LFP response in the cortex. *Arrowhead*: forepaw stimulus onset. **(B)** Representative LFP signals in response to forepaw stimulation before (black) and after (red) high-intensity a-tPCS. **(C)** LFP slope is quantified for the interval covering 20–80% of the negative deflection (denoted in red). **(D)** Time course of LFP slopes before and after low-intensity a-tPCS in the ACSF (*n* = 8) and prazosin (*n* = 4) condition. Gray area indicates a-tPCS application. **(E)** Time course of LFP slopes before and after high-intensity a-tPCS. **(F)** The LFP response is enhanced by low-intensity a-tPCS (*n* = 8) and decreased by high-intensity a-tPCS (*n* = 8). Moreover, the enhancement of LFP in the prazosin condition was decreased than that in the ACSF condition. Data of **(D–F)** were normalized to the baseline slope which was calculated by averaging slope values in 30 min before a-tPCS. Statistical significance was tested with Mann–Whitney test, which was implemented based on the non-normalized data, ^∗^*P* < 0.05. Error bars indicate SEM.

The a-tPCS has been proposed as a novel neuromodulatory tool to induce cortical plasticity in humans (Dissanayaka et al., 2017). Here, we explored the a-tPCS -induced cortical plasticity via LFP in the somatosensory cortex elicited by electrical forepaw stimulation in mice. We focused on the effects of different current intensities on the sensory plasticity (Figure 8). The LFP responses to forepaw stimulation were recorded on layer 2/3 of the contralateral somatosensory cortex. Figure 8A showed representative LFP traces in response to forepaw stimulation before 0.1 mA a-tPCS and at 50 min after a-tPCS. Figure 8B displayed representative LFP traces in response to forepaw stimulation before 0.35 mA a-tPCS and at 50 min after a-tPCS. The onset of the LFP response occurred about 8–12 ms after forepaw stimulation. The slope of the LFP was calculated based on the interval within 20 to 80% of the peak-to-peak amplitude of the initial negative deflection (Figure 8C). Normalized slopes were shown in Figures 8D,E. Before a-tPCS, the slopes of the LFP responses were stable during the control period of 30 min. After 0.1 mA a-tPCS, the slope of the LFP response gradually increased and remained potentiated throughout the recording period of 2 h (Figure 8D). On the contrary, 0.35 mA a-tPCS induced a decrease of slope of the LFP response (Figure 8E). Further analysis revealed that the mean LFP slope over 2 h increased by 36.2 ± 5.7% (Mann–Whitney test, *P* = 0.005, *n* = 8) after 0.1 mA a-tPCS and the mean LFP slope over 2 h decreased by 39.1 ± 3.9% (Mann–Whitney test, *P* = 0.036, *n* = 8) after 0.35 mA a-tPCS (Figure 8F). The a-tPCS-induced enhancement of LFP was decreased in the prazosin condition than that in the ACSF condition (Mann–Whitney test, *P* = 0.033, *n* = 4 for prazosin and *n* = 8 for ACSF) (Figures 8D,F).

## Discussion

In the present study, we directly compared the cortical responses evoked by a-tPCS with different current intensities in mouse somatosensory cortex. Two-photon calcium imaging revealed that 5 min a-tPCS with low-intensity current (0.1 mA) mainly activated astrocytes, whereas 5 min a-tPCS with high-intensity current (0.35 mA) activated both neurons and astrocytes. The astrocytic Ca^2+^ elevations are driven by noradrenergic-dependent activation of the A1ARs. The intense Ca^2+^ responses of neurons are driven by action potentials. By using electrophysiological recording, we found 5 min a-tPCS with low-intensity current could enhance LFP response while high-intensity a-tPCS decreased LFP response. Our results suggest that calcium elevation in astrocytes may play a role in a-tPCS-induced enhancement of cortical excitability, however, excessive calcium activity in neurons may diminish it.

Transcranial pulsed current stimulation has been reported to have positive effects on motor skills and cognitive function (Alon et al., 2012; Morales-Quezada et al., 2015). This method has become a potential neuromodulatory tool to treat neurological and psychiatric diseases, however, the cellular mechanisms of a-tPCS-induced cortical plasticity remain poorly understood. Calcium ions in neurons and astrocytes play crucial signaling roles in activity-dependent synaptic plasticity. Nonetheless, to our knowledge the calcium activities in cortical astrocytes evoked by a-tPCS have never been investigated systematically. In this study, we first explored the cortical response evoked by short-duration (2 s) a-tPCS. The stimulation frequency (2 Hz) and pulse width (450 ms) used in our experiments were similar to the stimulation parameters used in human studies (Jaberzadeh et al., 2014, 2015). We found that 0.35 mA a-tPCS could induce significant Ca^2+^ response in large numbers of neurons, however, significant Ca^2+^ response was not observed in neurons by 0.1 mA a-tPCS. Therefore, the threshold of current intensity to evoke detectable Ca^2+^ response in most neurons is above 0.1 mA. This result was in agreement with a previous report that the activation of cortical neurons was undetectable by using 0.1 mA tDCS (Monai et al., 2016). Our results also showed that the neurogliopil always had significant Ca^2+^ response to 2 s a-tPCS when the current intensity was ranged from 0.1 to 0.35 mA. Some studies using electrical microstimulation also showed the activation of neurogliopil (Histed et al., 2009; Michelson et al., 2019). Another important finding in our results is that 2 s a-tPCS cannot induce calcium response in astrocytes. Histed et al. (2009) investigated the neuronal and astrocytic responses to electrical microstimulation in mouse visual cortex. Their results suggested that neurons showed significant response, while astrocytes showed no fluorescence changes.

In practice, long-duration tPCS was applied over the cortex to alter cortical excitability. Therefore, we mainly studied the cortical response evoked by long-duration a-tPCS. Compared to the 2 s a-tPCS, 5 min a-tPCS with current intensity of 0.1 mA can elicit large-amplitude Ca^2+^ responses in astrocytes. The astrocytic activation occurred not only in neuron-dominant layer 2/3, but also in astrocyte-dominant layer 1. Moreover, the Ca^2+^ responses in 91.4% of neurons were undetectable. These results implied that the astrocytic Ca^2+^ surges might be driven in large part by direct electrical stimulation, rather than neuronal activity. In addition, astrocytes tightly ensheath neuronal somas, axons, dendrites, and synapses (Chung et al., 2015). In this study, the neurogliopil response to low-intensity a-tPCS was always reliably detected. The neurogliopil consists of glial processes, dendrites and axonal processes. A simulation study shows that axon terminals are more susceptible to polarization than pyramidal neuron somas during electrical stimulation (Rahman et al., 2013). Considering the axonal elements of the neurogliopil, the activation of axon terminals was possibly involved in astrocytic response to a-tPCS.

The results from LFP recording demonstrated that 5 min a-tPCS with 0.1 mA intensity could increase LFP response evoked by electrical forepaw stimulation. In our experiments, the enhancement of LFP response was also observed when the current intensity was increased to 0.15 mA. The enhancement of LFP was decreased by the application of prazosin. Calcium imaging showed small-amplitude Ca^2+^ response in some neurons and large-amplitude Ca^2+^ response in astrocytes. These results suggested that the calcium elevation in astrocytes may play an important role in the enhancement of LFP response following a-tPCS. In our experiments, responses of astrocytes 1 and 2 in Figure 3 appeared synchronized and quite similar. Given the close proximity of the two astrocytes, it is possible that there was a spread of Ca^2+^ wave from one astrocyte to neighboring astrocyte. The Ca^2+^ waves in astroglial networks may modulate neuronal network activity (Paixao and Klein, 2010). In our study, the a-tPCS-induced astrocytic Ca^2+^ elevations are driven by noradrenergic-dependent activation of the A1ARs. The A1ARs participate in Ca^2+^-signaling in astrocytes and activate the release of ATP and D-serine from astrocytes (Pankratov and Lalo, 2015). The release of ATP and D-serine from astrocytes can modulate the long-term synaptic plasticity (Araque et al., 2014; Lalo et al., 2014). The enhanced LFP response following low-intensity a-tPCS might be partly due to activation of astrocytic A1ARs. Our results were similar to the results from a previous study, which demonstrated that tDCS-induced plasticity was associated with Ca^2+^ surges in astrocytes (Monai et al., 2016). This is reasonable, as the a-tPCS is an unbalanced current with some degrees of net direct current component (Jaberzadeh et al., 2014). a-tPCS alters cortical excitability by a combination of tonic and phasic effects, the application of prazosin may block the tonic effects. In this study, the short inter-pulse interval of a-tPCS could result in a large direct-current component.

In our study, large-amplitude Ca^2+^ responses in neurons and astrocytes were evoked by 5 min a-tPCS with current intensity of 0.35 mA. Statistical analysis indicated that the amplitudes of Ca^2+^ responses in neurons and astrocytes evoked by 0.35 mA a-tPCS were significantly higher than those evoked by 0.1 mA a-tPCS, respectively. In addition, the response onset of the astrocytic Ca^2+^ surge evoked by 0.35 mA a-tPCS was significantly earlier than that evoked by 0.1 mA a-tPCS. These results demonstrated that high-intensity a-tPCS can induce intense calcium changes in large populations of cells within a short time. In Figure 5C, neurons exhibit initially high calcium response, which is followed by a period of lower activity throughout the rest of the stimulation period. In Figure 7A, some neurons show the similar temporal activation pattern, while others exhibit a rapid plateau during electrical stimulation. These two different activation patterns have been reported in a recent study using continuous pulsed current stimulation (Michelson et al., 2019). The temporal activation pattern of most neurons in our study is similar to that of the onset neurons, which are distal to the stimulation electrode in their study. In our study, the imaging field of view is distal from the stimulation electrode. The activation patterns of neurons in this study are different from those of neurons in a previous study (Monai et al., 2016). The variances may be due to differences in stimulation modes (continuous pulsed current stimulation vs. direct current stimulation) and current intensities (0.35 mA vs. 0.1 mA).

To this date, this is the first study investigating the effects of a-tPCS in an animal model. The effects of low-intensity a-tPCS in this study were in accordance with the results reported by previous studies in humans, which showed that 1.5 mA a-tPCS could induce a significant increase in the amplitude of motor evoked potential (Jaberzadeh et al., 2014, 2015). The finding that a-tPCS with high-intensity led to a reduction in cortical excitability was seldom reported. On the contrary, Morales-Quezada et al. (2014) showed that the high-intensity stimulation resulted in stronger effects while the low-intensity stimulation did not produce significant effects compared with sham stimulation. However, larger current intensity may not always result in stronger effects. For example, Bastani and Jaberzadeh (2013) reported that the tDCS with smallest current intensity of 0.3 mA produced larger corticospinal excitability changes than the two higher current intensities of 0.7 mA and 1.4 mA (Bastani and Jaberzadeh, 2013). An increase of stimulation intensity does not necessarily enhance the efficacy of stimulation, but might even shift the direction of excitability alterations (Batsikadze et al., 2013). The results of the present study suggest that current intensity is a critical parameter in the application of a-tPCS. Besides, prolongation of stimulation duration may also result in reverse effects (Batsikadze et al., 2013; Monte-Silva et al., 2013). For example, doubling the stimulation duration from 13 to 26 min led to inhibitory aftereffects, probably due to a calcium overflow-caused neuronal counter-regulation (Monte-Silva et al., 2013). In our study, the key difference between responses at high and low intensity is that 0.35 mA a-tPCS induced synchronized and intense Ca^2+^ response in a large population of neurons. The neuronal response was blocked by the application of TTX, suggesting that the intense Ca^2+^ responses of neurons are driven by action potentials. The depolarization of neurons induces an influx of intracellular calcium. The calcium influx can cause Ca^2+^ release from the endoplasmic reticulum (Sharp et al., 1993). However, excessive elevations in intracellular Ca^2+^ could lead to a calcium-dependent neurodegeneration in excitotoxicity (Arundine and Tymianski, 2003). By using a mouse model of ischemia, Murphy et al. (2008) found that increases in intracellular calcium levels could lead to dendritic damage and spine loss after 2–3 min of global ischemia. The damage to dendrites and spines would result in decreased neuronal activity. Power spectrum analysis indicated that the average EEG power within the first 20 min was decreased to about 2% of preischemic values. Therefore, the decreased LFP response following 0.35 mA a-tPCS might be associated with intense Ca^2+^ response of neurons. Hence, our results may provide some indication for the use of tPCS in animal and human studies.

The calcium response can be reliably detected in neurogliopil region, which contains many fine processes of the surrounding neurons and astrocytes. In these places, astrocyte processes closely interact with neuronal synapses and modulate synaptic transmission and plasticity (Araque et al., 2014; Chung et al., 2015; Haydon and Nedergaard, 2015; Oliveira et al., 2015). The brain function arises from the coordinated activity of neuron-glia networks (Perea et al., 2014). Many studies reported that tPCS can modulate brain oscillatory activity and enhance functional connectivity (Guleyupoglu et al., 2013; Fitzgerald, 2014; Jaberzadeh et al., 2014). Due to the different recording methods, the calcium activity observed in our experiments cannot be directly compared with their results, which were mainly based on EEG recording. Although the role of neurogliopil Ca^2+^ response in the modulation of cortical oscillations and functional connectivity is unclear, we believe that the Ca^2+^ response of neurogliopil would influence the activity of neuronal networks. In short, we cannot rule out the possibility that the neurogliopil response may also play a role in a-tPCS-induced plasticity.

The findings in this study should be considered in the context of several limitations. Firstly, we acknowledge that the IP_3_R2 (inositol trisphosphate receptor type 2) knockout mice should be used to prove the involvement of astrocytic GPCR (G-protein-coupled receptor) activation (Monai et al., 2016) in mediating a-tPCS-induced effects on LFP. There is no direct evidence of an involvement of astrocytic Ca^2+^ signaling in mediating a-tPCS-induced effects on LFP due to the lack of experiments in IP_3_R2 knockout mice. However, the pharmacological experiments using prazosin demonstrated the involvement of noradrenergic activation of A1ARs, which transduce the G_q_ signaling cascade for production of IP_3_. Secondly, the neuropil response to sensory stimulation after tDCS was enhanced in layer 2/3 of the cortex, but not in layer 4 where sensory thalamic input arrives (Monai and Hirase, 2016; Monai et al., 2016). Some related studies have demonstrated that synaptic plasticity in layer 4 disappears as the animal matures after the critical period (Daw et al., 1992; Fox, 1992). In this study, the depth of imaging is limited to the superficial layers (layers 1–3) of the cortex. Therefore, the findings in the current study are only pertinent to the superficial layers of the cortex. Thirdly, the anodal current stimulation generally enhances cortical excitability. However, the a-tPCS involves the injection of much monopolar current. Compared to a-tPCS, transcranial alternating current stimulation (tACS) is a balanced current consisting of bipolar alternating current with equal electric charge. No irreversible electrochemical products are known to accumulate at the electrode (Antal et al., 2017). When considering translational validity in humans, a balanced tACS protocol might be an alternative. In short, the a-tPCS parameters in our study should be adjusted carefully when directly translated into the clinical conditions.

## Conclusion

In summary, the calcium response in mouse somatosensory cortex evoked by long-duration a-tPCS was reported for the first time. Low-intensity a-tPCS elicited large-amplitude Ca^2+^ response in astrocytes but not in neurons. High-intensity a-tPCS elicited large-amplitude Ca^2+^ responses in both neurons and astrocytes. The enhancement of cortical excitability induced by low-intensity a-tPCS might be partly associated with astrocytic Ca^2+^ elevations, which is dependent on noradrenergic activation of A1ARs. The decrease of cortical excitability induced by high-intensity a-tPCS may be caused by excessive calcium activity in neurons. These findings would contribute to the understanding of mechanisms underlying a-tPCS-induced cortical plasticity, and also suggest that the appropriate current intensity should be used in the application of a-tPCS.

## Data Availability

All datasets generated for this study are included in the manuscript and/or the supplementary files.

## Ethics Statement

Animal Subjects: The animal study was reviewed and approved by Animal Care and Use Committee of the Tianjin Medical University.

## Author Contributions

ZM and HS designed the experiments. ZM, FW, and YL conducted the experiments. ZM, XD, RD, and AL analyzed the data. ZM and HS wrote the manuscript. LW, SH, FC, QH, CG, QJ, SL, and BF participated in discussion. All authors reviewed the manuscript.

## Conflict of Interest Statement

The authors declare that the research was conducted in the absence of any commercial or financial relationships that could be construed as a potential conflict of interest.
